# Editorial: Insights in social psychiatry and psychiatric rehabilitation: 2023

**DOI:** 10.3389/fpsyt.2024.1463602

**Published:** 2024-08-12

**Authors:** Antonio Vita, Jacopo Lisoni

**Affiliations:** ^1^ Department of Clinical and Experimental Sciences, University of Brescia, Brescia, Italy; ^2^ Asst Spedali Civili Brescia - Department of Mental Health, Brescia, Italy

**Keywords:** social psychiatry, psychiatric rehabilitation, treatment outcomes, recovery, severe mental illness

This Research Topic spans on several crucial areas of Social Psychiatry and Psychiatric Rehabilitation. The need to provide updated and innovative models of care for people living with severe mental illness (SMI) is discussed in Killaspy et al. and in Harvey et al. The provision of integrated psychological approaches in rehabilitative contexts is discussed in Melicherova et al. Ferentinos et al., and Rexhaij et al. argued on the need to further promote advocacy and quality of care through psychoeducational programs for both family members and mental health workers. Van der Post et al. investigated the effects of coercive measures on treatment outcomes. Finally, Scognamiglio et al. presented insightful data on the role of defensive medicine among psychiatrists.

The opinion paper by Killaspy et al. clearly described the mission of mental health rehabilitation services in the achievement of recovery for patients with SMI. Covering current evidence on the effectiveness, feasibility, and limitations of mental health rehabilitation services in UK, this paper also examines some issues to promote the research in the field (e.g., the effectiveness of rehabilitation services for people at an early stage of psychosis, the role of peer support in rehabilitation services, etc.) with the aim to further improve the care of people living with SMI.

Harvey et al. performed a systematic review, with 59 included studies, aimed to generate a narrative synthesis of recent evidence on the effectiveness of community-based models of care (MoCs) to support clinical, functional, and personal recovery outcomes of people living with persistent and complex mental health needs. Beneficial MoCs ranged from well-established to novel and updated models and those explicitly addressing recovery goals, but the authors found that established MoCs, such as intensive case management, are among the most effective practices supporting clinical and functional recovery. Despite these positive findings, the authors also highlighted the urgent need to provide continue evolution and adaptation of MoCs, suggesting that further attention to service innovation and research is required.

Considering the need to provide integrated treatments for depressive disorders, Melicherova et al. performed a quasi-randomized study aimed at comparing the efficacy of Behavioral Activation (BA) compared to Cognitive-Behavioral Therapy (CBT) when embedded in inpatient psychosomatic rehabilitation treatment. Indeed, BA was considered a viable alternative to CBT in clinical settings, especially when large groups of patients need to be treated in relatively condensed ways. In 375 inpatients randomly assigned to either BA or CBT, it was found that both groups showed substantial reductions in depressive symptoms, response rates and functioning improvement at the end of treatment and at follow-ups. These results clearly demonstrate the effectiveness of short-term inpatient psychotherapy programs for the treatment of depression within the context of psychosomatic rehabilitation. Moreover, as both psychological approaches were associated to significant improvement, the authors underlined the feasibility of BA in rehabilitative clinics, considering its lower requirements of cognitive abilities and its easier implementation, suggesting that BA could be an effective psychotherapeutic alternative.

Considering the role of expressed emotion (EE) in different care setting, Ferentinos et al. investigated patient- and caregiver-related predictors of EE toward individuals with schizophrenia in families and halfway houses. Conducted on 40 individuals with schizophrenia living with their families (“outpatients”) and 40 “inpatients” in halfway houses, the study recorded the EE of 56 parents or 22 psychiatric nurses, respectively. Findings from this study provided setting specific pathogenetic pathways of emotional overinvolvement (EOI): for example, it was found that nurses displayed higher EOI when older or less experienced and lower EOI toward inpatients with more severe negative symptoms. These findings suggest the need to provide psychoeducational interventions for professional caregivers to improve their caregiving capacity, for example, by improving understanding of negative symptoms and by coping strategies toward these symptoms.

Considering the vital role of informal caregivers in the treatment and support of close relatives with SMI, Rexhaij et al. performed a single-center RCT to assess the efficacy of the Ensemble program (a brief individual intervention designed to support informal caregivers), compared to support as usual, on 149 healthy participants with a high care burden. It was found that the Ensemble program could represent a suitable recovery-based approach, able to significantly reduce distress and burden and to increase optimism among informal caregivers.

van der Post et al. investigated the effects of coercive measures on treatment outcomes, assessed with the Health of the Nation Outcome Scales (HoNOS), in 786 involuntarily admitted patients in Netherlands. As more than two-fifths of the involuntarily admitted patients were subjected to coercive measures, the authors found that HoNOS scores in the group without coercion improved by nearly two and a half points more on average than those of the group with coercion. However, despite patients with coercion had lower improvements in HoNOS scores, the authors concluded that a causal relationship between the use of coercion and less favorable treatment outcomes could neither be concluded nor ruled out.

In this national survey on 256 Italian psychiatrists, Scognamiglio et al. argued on the fact that defensive medicine is a common phenomenon among psychiatrists due to their “position of guarantee”. Indeed, the survey evaluated the attitude and behaviors on defensive medicine and professional liability suggesting that most psychiatrists, especially the youngest, reported to practice defensive medicine. Moreover, psychiatrists considered that the position of guarantee compromised the quality of care for their patients. Furthermore, acute and high-intensity medical care settings were associated with more frequent malpractice claims and complaints, as well as with greater propensity to act defensively. The authors also found that, despite most psychiatrists were concerned about civil and criminal laws on professional responsibility, many were not fully informed about recent legislative regulations and younger physicians resulted scarcely trained in risk management. Thus, it was suggested that education on legal implications and risk management should be further provided to reduce defensive practices among psychiatrists to improve the quality of care.

In conclusion, we believe that the new, updated and captivating data presented in this Research Topic very contribute to increase our knowledge in the field of Social Psychiatry and Psychiatric Rehabilitation, further promoting and improving the care of people suffering from SMI.

